# The Treatment of the Complications

**Published:** 1892-12-17

**Authors:** 


### The Treatment of the Complications.

*Diarrhœa.*—This is generally treated with castor oil. The mixture used at Great Ormond Street is made up as follows: Castor oil m v., mucilage of acacia m. lv., peppermint water ad ʒj. It is also advisable to treat the irritable stomach condition which is generally present with a mixture composed of bicarbonate of soda and some bismuth with an aromatic. The following mixture is a popular one at this hospital: Bicarbonate of soda gr. ii., aromatic spirit of ammonia m. ii., glycerine m. v., peppermint water to ʒj. It is also important that the abdomen and chest should be protected by woollen garments. Should the measures above indicated fail to check the diarrhœa, one to two grains of grey powder with the same quantity of Dover's powder should be tried, and repeated according to the effect produced.

*Bronchitis.*—It is essential that bronchitis should be at once treated, however slight. The treatment should be conducted on general principles.

*Convulsions and Laryngismus.*—These may be relieved by small doses of chloral and bromide, the dose being regulated according to the age of the child. Cold or tepid sponging two or three times a day is also found to be of service.

